# Cost-utility analysis of molnupiravir for high-risk, community-based adults with COVID-19: an economic evaluation of the PANORAMIC trial

**DOI:** 10.3399/BJGP.2023.0444

**Published:** 2024-07-09

**Authors:** May Ee Png, Victoria Harris, Jenna Grabey, Nigel D Hart, Bhautesh D Jani, Daniel Butler, Andrew Carson-Stevens, Maria Coates, Lucy Cureton, Melissa Dobson, Jienchi Dorward, Philip Evans, Nick Francis, Oghenekome A Gbinigie, Gail Hayward, Jane Holmes, Kerenza Hood, Saye Khoo, Haroon Ahmed, Mark Lown, Micheal McKenna, Sam Mort, Jonathan S Nguyen-Van-Tam, Najib M Rahman, Duncan B Richards, Nicholas PB Thomas, Oliver van Hecke, Richard Hobbs, Paul Little, Ly-Mee Yu, Christopher C Butler, Stavros Petrou, on behalf of the PANORAMIC Trial Collaborators

**Affiliations:** Nuffield Department of Primary Care Health Sciences, University of Oxford, Oxford, UK.; Nuffield Department of Primary Care Health Sciences, University of Oxford, Oxford, UK.; Nuffield Department of Primary Care Health Sciences, University of Oxford, Oxford, UK.; Dentistry and Biomedical Sciences, Queen’s University Belfast, UK.; General Practice and Primary Care, School of Health and Wellbeing, College of Medical, Veterinary & Life Sciences, University of Glasgow, Glasgow; Dentistry and Biomedical Sciences, Queen’s University Belfast, UK.; Division of Population Medicine, School of Medicine, Cardiff University, Cardiff, UK.; Nuffield Department of Primary Care Health Sciences, University of Oxford, Oxford, UK.; Nuffield Department of Primary Care Health Sciences, University of Oxford, Oxford, UK.; Oxford Respiratory Trials Unit, Nuffield Department of Medicine, University of Oxford, Oxford, UK.; Nuffield Department of Primary Care Health Sciences, University of Oxford, Oxford, UK; Centre for the AIDS Programme of Research in South Africa (CAPRISA), University of KwaZulu–Natal, Durban, South Africa.; Faculty of Health and Life Sciences, University of Exeter, Exeter, UK; National Institute for Health and Care Research (NIHR) Clinical Research Network, University of Leeds, Leeds, UK.; Primary Care Research Centre, University of Southampton, Southampton, UK.; Nuffield Department of Primary Care Health Sciences, University of Oxford, Oxford, UK.; Nuffield Department of Primary Care Health Sciences, University of Oxford, Oxford, UK.; Nuffield Department of Primary Care Health Sciences, University of Oxford, Oxford, UK.; Centre for Trials Research, Cardiff University, Cardiff, UK.; Department of Pharmacology, University of Liverpool, Liverpool, UK.; Division of Population Medicine, School of Medicine, Cardiff University, Cardiff, UK.; Primary Care Research Centre, University of Southampton, Southampton, UK.; Nuffield Department of Primary Care Health Sciences, University of Oxford, Oxford, UK.; Nuffield Department of Primary Care Health Sciences, University of Oxford, Oxford, UK.; Lifespan and Population Health Unit, School of Medicine, University of Nottingham, Nottingham, UK.; Oxford Respiratory Trials Unit, Nuffield Department of Medicine, University of Oxford, Oxford; Oxford NIHR Biomedical Research Centre, Oxford; Chinese Academy of Medicial Sciences Oxford Institute, University of Oxford, Oxford, UK.; Nuffield Department of Orthopaedics, Rheumatology and Musculoskeletal Sciences, University of Oxford, Oxford, UK.; Witney; NIHR Thames Valley and South Midlands Clinical Research Network, UK; Royal College of General Practitioners, London, UK.; Nuffield Department of Primary Care Health Sciences, University of Oxford, Oxford, UK.; Nuffield Department of Primary Care Health Sciences, University of Oxford, Oxford, UK.; Primary Care Research Centre, University of Southampton, Southampton, UK.; Nuffield Department of Primary Care Health Sciences, University of Oxford, Oxford, UK.; Nuffield Department of Primary Care Health Sciences, University of Oxford, Oxford, UK.; Nuffield Department of Primary Care Health Sciences, University of Oxford, Oxford, UK.

**Keywords:** antiviral drugs, cost-benefit analysis, COVID-19, molnupiravir, quality-adjusted life years, SARS-CoV-2

## Abstract

**Background:**

The cost-effectiveness of molnupiravir, an oral antiviral for early treatment of SARS-CoV-2, has not been established in vaccinated populations.

**Aim:**

To evaluate the cost-effectiveness of molnupiravir relative to usual care alone among mainly vaccinated community-based people at higher risk of severe outcomes from COVID-19 over 6 months.

**Design and setting:**

An economic evaluation of the PANORAMIC trial in the UK.

**Method:**

A cost-utility analysis that adopted a UK NHS and personal social services perspective and a 6-month time horizon was performed using PANORAMIC trial data. Cost-effectiveness was expressed in terms of incremental cost per quality-adjusted life year (QALY) gained. Sensitivity and subgroup analyses assessed the impacts of uncertainty and heterogeneity. Threshold analysis explored the price for molnupiravir consistent with likely reimbursement.

**Results:**

In the base-case analysis, molnupiravir had higher mean costs of £449 (95% confidence interval [CI] = 445 to 453) and higher mean QALYs of 0.0055 (95% CI = 0.0044 to 0.0067) than usual care (mean incremental cost per QALY of £81 190). Sensitivity and subgroup analyses showed similar results, except for those aged ≥75 years, with a 55% probability of being cost-effective at a £30 000 per QALY threshold. Molnupiravir would have to be priced around £147 per course to be cost-effective at a £15 000 per QALY threshold.

**Conclusion:**

At the current cost of £513 per course, molnupiravir is unlikely to be cost-effective relative to usual care over a 6-month time horizon among mainly vaccinated patients with COVID-19 at increased risk of adverse outcomes, except those aged ≥75 years.

## Introduction

Globally, the COVID-19 pandemic has infected >676 million people and resulted in >14.9 million excess deaths between 2020 and 2021.^1^^,^^2^ It has also had an adverse impact on economies worldwide as a result of public health measures and social distancing to mitigate the spread of COVID-19.^3^ In particular, the UK reported a record fall in real Gross Domestic Product of nearly 10% in 2020, which was greater than most advanced economies in Europe and North America.^3^ Furthermore, persisting symptoms that arise from COVID-19 and last ≥4 weeks after acute infection have adversely affected the day-to-day activities of 1.5 million people in the UK, with 20% being ‘limited a lot’ in their day-to-day activities.^4^

Although several COVID-19 vaccines are highly effective in reducing the incidence of serious consequences of COVID-19, namely admission to hospital and death,^5^^,^^6^ they cannot eliminate the disease, and evidence from previous studies^7^^–^^9^ has highlighted the need to initiate treatment for COVID-19 with antivirals/antibodies as soon as possible after the onset of symptoms. It also suggests that the treatment should ideally be *‘readily available and easily administered by the patients themselves’* in the community.^10^

Molnupiravir is a small-molecule ribonucleoside prodrug of N-hydroxycytidine with direct antiviral activity against SARS-CoV-2 and other RNA viruses, and was approved in the UK for emergency use in November 2021 for the treatment of COVID-19.^11^ Previous studies^10^^,^^12^ have examined the clinical effectiveness of molnupiravir in patients not admitted to hospital, where molnupiravir was found to reduce the risk of admission to hospital or death in at-risk, unvaccinated adults with COVID-19^10^ but not among a mainly vaccinated population with COVID-19.^12^ Its cost-effectiveness remains undetermined in this population, to the authors’ knowledge. Therefore, this study aimed to compare the cost-effectiveness of molnupiravir plus usual care versus usual care alone among community-based people at high risk of more severe COVID-19 outcomes, using data from the Platform Adaptive trial of NOvel antiviRals for eArly treatMent of COVID-19 In the Community (PANORAMIC) trial.

**Table:** How this fits in

Previous cost-effectiveness analyses of molnupiravir versus usual care among mostly unvaccinated populations had mixed conclusions. To the authors’ knowledge, no trial-based economic evaluations of molnupiravir have been published. Molnupiravir, priced at £513 per course, is unlikely to be cost-effective compared with usual care from the UK NHS and personal social services perspective over 6 months for high-risk, community-based adults with COVID-19. However, it could be cost-effective at £30 000 per quality-adjusted life year for those aged ≥75 years. It has to cost one-third of the current market price to be cost-effective from this perspective. These findings will inform procurement strategies and influence policy on antiviral treatments for COVID-19.

## Method

### Background of trial

The PANORAMIC trial (ISRCTN30448031) was a national, multicentre, primary care, open-label, multigroup, prospective, platform adaptive trial of early treatments for COVID-19 in the UK, which has a national health service that provides publicly funded health care, primarily free of charge at the point of use. Full details of the clinical trial including its sample size requirements, sampling procedures, and clinical outcomes are published elsewhere.^12^

In brief, the participants included were people in the community (that is, not in hospital) aged ≥50 years (or ≥18 years with relevant comorbidities) who had COVID-19 symptoms that had started within the past 5 days, and had a positive polymerase chain reaction or rapid antigen SARS-CoV-2 test within the past 7 days. Participants were randomly assigned on a 1:1 basis to receive oral 800 mg molnupiravir twice daily for 5 days plus usual care or usual care only. The study was unblinded (no placebo control), and evaluated molnupiravir from 8 December 2021 to 27 April 2022, by which time 98.9% (*n* = 25 508/25 783) of participants had been vaccinated at least once, with a mode of three vaccine doses per vaccinee.

The Consolidated Health Economic Evaluation Reporting Standards 2022 guidelines^13^ have been followed when reporting this health economic evaluation, in a format appropriate to stakeholders and policymakers.

### Measurement of resource use

Resource-use data were collected through two main sources. First, trial data were obtained from participants by online daily diaries completed over the first 28 days post-randomisation, and online questionnaires were completed by participants at 3 and 6 months post-randomisation. The online questionnaires reported resource use between 28 days and 3 months post-randomisation, and subsequently between 3 and 6 months post-randomisation. Non-responders were telephoned on days 7, 14, and 28, as well as at months 3 and 6, where applicable. Second, routine electronic healthcare data extracted from national routine electronic healthcare databases were used, including Hospital Episode Statistics for England (April 2023 dataset), the Secure Anonymised Information Linkage Databank for Wales (March 2023 dataset), the electronic Data Research and Innovation Service for Scotland (January 2023 dataset), and data provided by the HSC Business Services Organisation Honest Broker Service in Northern Ireland (May 2023 dataset).

The type and frequency of use of primary care (that is, GP, practice nurse, NHS 111, ambulance service, community nurse, physiotherapist, counsellor, social worker, home carer, and occupational therapist) and secondary care (that is, hospital admission, emergency care, and hospital respiratory outpatient clinic) services because of symptoms associated with COVID-19 was recorded in the daily diaries and trial questionnaires. Secondary care resource-use data collected as part of the participant-completed research instruments were complemented and validated by data extracted from national routine electronic healthcare databases from each of the UK nations. Participants also recorded time off work because of symptoms associated with COVID-19 in the 3-and 6-month follow-up questionnaires.

Where there was a divergence of resource-use estimates extracted from alternative data sources, the following hierarchy for selecting the preferred source of resource-use data was adopted. For admissions to hospital, the primary data source was participant-reported data recorded in the trial admission to hospital case-report form whereas the secondary data source came from the routine electronic healthcare datasets. This approach, which mirrored the approach adopted by the trial’s master statistical analysis plan (MSAP), was chosen because of a data reporting lag in the routine electronic healthcare databases as observed in a similar trial (that is, the PRINCIPLE trial^14^). Furthermore, a comprehensive review of self-reported utilisation of healthcare services by Bhandari and Wagner^15^ noted that *‘respondents had better recall for major events such as hospitalisations versus physician visits’* when self-reported data were compared with data reported in health records. As it was also noted in this review that *‘self-report accuracy increases for inpatient visits compared to outpatient visits’*,^15^ routine healthcare data were the primary source for all hospital-related resource use except admissions to hospital where participant-reported data were the primary source. As the routine healthcare data did not capture non-hospital resource use (that is, community-related resource use), only participant-reported data were used in the current analysis for these resource categories.

### Valuation of resource use

All resource-use estimates were valued in monetary terms using the latest and most appropriate UK unit costs or participant valuations estimated at the time of analysis (Supplementary Table S1). Adjustments were made for inflation to financial year 2020/2021 prices using the Personal Social Services Research Unit (PSSRU) Hospital & Community Health Services Index^16^ where applicable. The purchase price of molnupiravir (at £513 per course) was obtained from publicly available data.^17^ NHS reference costs^18^ were employed to value hospital resource use (for example, inpatient visits that included day cases and longer stays [that is, elective and non-elective admissions], emergency department visits, and outpatient attendances) and the PSSRU Unit Costs of Health and Social Care^16^ compendium was used to value community health and social service resource inputs. The costs for each hospital event extracted from the routine datasets were estimated by linking the Healthcare Resource Group codes for each inpatient and day case admission, outpatient attendance, and accident and emergency visit with NHS reference costs^18^ for the financial year 2020/2021.

Unit costs of medications were obtained from the Prescription Cost Analysis database.^19^ The median national wage obtained from the Office for National Statistics^20^ was used for the valuation of participants’ work losses.

### Measurement of outcomes

The primary measure of health consequence was the quality-adjusted life year (QALY) derived from utility scores that were obtained using the EQ-5D-5L health-related quality-of-life instrument.^21^ The EQ-5D-5L instrument facilitates the generation of a utility score that reflects the value of a person’s health-related quality of life on a cardinal scale where zero represents death and one represents full health. A utility score refers to the preference value for any particular set of health outcomes. The EQ-5D-5L descriptive system consists of five health dimensions (mobility, self-care, usual activity, pain/discomfort, and anxiety/depression) each with five levels of health status to choose from (no problems, slight problems, moderate problems, severe problems, and extreme problems). The EQ-5D-5L also contains a visual analogue scale (VAS), which is a non-preference-based quantitative measure of health outcome that records a participant’s self-rated health on a scale indexed at zero, representing the worst health imagined, and 100 representing the best health imagined. EQ-5D-5L measurements were recorded using the daily diaries and trial questionnaires at baseline, 14 days, 28 days, and 3 and 6 months post-randomisation.

### Valuation of outcomes

Utility scores were derived from responses to the EQ-5D-5L descriptive system. UK utility values were derived using the approach recommended by the National Institute for Health and Care Excellence (NICE),^22^ which currently consists of applying a validated mapping function onto the UK EQ-5D-3L tariff set that has been developed by the NICE Decision Support Unit.^23^ For the primary analysis, QALYs were calculated as the area under the baseline-adjusted utility curve across each time point of assessment using the trapezoidal rule.^24^

### Data cleaning

Face validity tests were conducted on the study data (for example, to identify misspelt text) and checked against the source documents according to the data management plan. Records of resource use across different time points were also cross-checked to ensure that there was no duplication. Corrections made were documented in the statistical code. Free-text entries reported by patients in the ‘Others’ field of the resource-use questionnaires captured the use of other health and social services not listed as any of the options in the questionnaires. These resource inputs were cleaned and subsequently valued using the relevant unit costs described in the ‘valuation of resource use’ section.

### Missing data

Following the methodological guidance described by Faria *et al*^25^ to ascertain the nature and pattern of missing data, the data were treated as missing at random and the multiple imputation method was used to impute missing costs and utility scores. This was done using chained regression equations predicting missing values from the observed covariates (observed responses of participants) and creating sets of multiple datasets containing possible values for missing observations.^25^ Pooled estimates were then computed using Rubin’s rules^26^ to obtain overall mean estimates of the costs and utility scores per participant.

Mean imputation by treatment arm was used for missing baseline covariates. Multiple imputation for QALYs was performed at the individual utility score-level across the entire follow-up period. Multiple imputation for costs was performed at total cost-level (for example, mean total cost from the NHS and personal social services [PSS] perspective) for individual participants at each follow-up time point. The multiple imputation was performed using Amelia II in R (version 4.2).^27^ This multiple imputation package has been shown to outperform other packages such as NORM, MICE, and SPSS MI.^28^ Independent variables included in the imputation models consisted of treatment allocation and baseline covariates such as age at randomisation, sex, ethnicity, nation the participant was recruited from, smoking status, presence of comorbidities, presence of major symptoms, use of inhaled corticosteroids, vaccination status, swab positivity status, NHS priority category, and EQ-5D VAS score. This imputation was run 25 times according to the ‘rule of thumb’ that suggests that the number of imputations should be similar to the percentage of incomplete cases.^29^

### Data analysis

The base-case analysis included a within-trial analysis using imputed data that consisted of all randomised participants, which is in accordance with the ‘intention-to-treat’ principle, taking a 6-month time horizon from an NHS and PSS perspective. All the costs, except the values placed on lost productivity from time off work, were included in the base-case analysis under the NHS and PSS perspective. All analyses were carried out using R (version 4.2). The economic evaluation was prospectively planned and detailed within a ‘health economic analysis plan’ (HEAP). The HEAP was finalised and approved by the Trial Steering Committee before unblinding.

Costs and QALYs were not discounted to present values because the follow-up period was <1 year. Estimates of resource use were summarised by treatment allocation group and follow-up period, and differences between groups were analysed using *t*-tests for continuous variables and Pearson’s χ^2^-test for categorical variables. Means and standard errors (SEs) for values of each cost category were estimated by treatment allocation and follow-up period. Mean differences in total costs and utility scores between the treatment arms were estimated using *t*-tests and bootstrap 95% confidence intervals (CIs) that were computed based on 10 000 replications. The bootstrap used Monte Carlo simulations to resample datasets based on the original data. A two-sided significance level of 0.05 was used throughout.

Cost and QALY data were combined to calculate incremental cost-effectiveness ratios (ICERs) and net monetary benefit (NMB) statistics from the NHS and PSS perspective in the base-case analysis. A seemingly unrelated regression model was fitted to the imputed data to estimate total costs and total QALYs in each treatment arm over the 6-month follow-up period. This approach allows for correlation between costs and outcomes, and estimates the two regression equations jointly, potentially improving the precision of the estimates. The model was adjusted using the stratification factors (that is, age, vaccination status, and comorbidity status). Incremental cost-effectiveness thresholds of £20 000 and £30 000 per QALY were used, as recommended by NICE.^30^ An additional £15 000 per QALY cost-effectiveness threshold was also included to reflect recent trends in healthcare decision making.^31^

### Uncertainty analysis

A non-parametric bootstrapping approach was used to determine the level of sampling uncertainty surrounding the mean ICER by generating 10 000 estimates of incremental costs and benefits. Decision uncertainty was characterised by estimating the probability that each treatment option was cost-effective at different cost-effectiveness thresholds, including the threshold values of £15 000 per QALY, £20 000 per QALY, and £30 000 per QALY described above, and displayed graphically using cost-effectiveness acceptability curves.

### Sensitivity analysis

Three sensitivity analyses were conducted in this study. First, the study perspective was broadened to a societal perspective that included economic values placed on lost productivity. Second, complete case analysis was used to assess the impact of missing data on the ICERs. Third, the price per treatment course of molnupiravir at which it should be recommended for reimbursement on cost-effectiveness grounds assuming incremental cost-effectiveness thresholds of £15 000, £20 000, and £30 000 per QALY were explored. The latter analysis adopted an NHS and PSS perspective, and used the same approaches to imputing missing data and accounting for correlation between costs and outcomes as the baseline analysis. The threshold analysis was conducted as there was no NHS indicative price for molnupiravir at the time of writing.

### Subgroup analysis

Subgroup analyses were conducted to explore potential heterogeneity in the incremental cost-effectiveness of molnupiravir. The subgroup analyses were specified a priori in accordance with the MSAP^12^ and the HEAP, as outlined in Supplementary Table S2.

A post hoc subgroup analysis of participants aged ≥75 years was also included as molnupiravir was found to be cost-effective relative to usual care among participants aged ≥75 years at an upper cost-effectiveness threshold of £30 000 per QALY. The key results are presented in the later sections of this article and the detailed results are presented in Supplementary Information S1, Supplementary Tables S3–S7, and Supplementary Figure S1.

### Patient and public involvement

The underpinning PANORAMIC trial involved patients and members of the public in a number of ways, including the refinement of the study question, and the design and implementation of patient-facing documents as described in the trial protocol.^32^ The authors also intend to disseminate the main results to trial participants and the public, and have sought the PANORAMIC trial’s patient and public involvement members in the interpretation and development of appropriate methods of dissemination.

## Results

### Completion rate of resource use and EQ-5D-5L

Between 8 December 2021 and 27 April 2022, 12 821 participants were randomised to molnupiravir with usual care and 12 962 participants were randomised to usual care alone. The baseline characteristics of the participants by treatment arm are summarised in Supplementary Table S8. The mean age of the participants was 56.7 years (SE 0.1) in the molnupiravir arm and 56.5 years (SE 0.1) in the usual care arm. Baseline characteristics were similar between the treatment arms. Participants were predominantly female (*n* = 15 099/25 783, 58.6%), had comorbidities (*n* = 17 759/25 783, 68.9%) and received at least three doses of a SARS-CoV-2 vaccine (*n* = 24 356/25 783, 94.5%).

A breakdown of completion rates by resource-use category (from an NHS and PSS perspective) and EQ-5D-5L measure from baseline to 6 months post-randomisation is presented in Supplementary Table S9. Of all the participants, there were 7822 (61.0%) in the molnupiravir arm and 6984 (53.9%) in the usual care arm that had complete resource-use and EQ-5D-5L data across all time points. The data were non-monotonic as a few participants with missing data at one follow-up time point had completed questionnaires at subsequent time points.

### Health and social care resource utilisation and time off work

In general, there were no statistically significant differences in health and social care resource utilisation between the treatment arms during each period of follow-up (Supplementary Table S10) in the available case analysis. The exceptions were that NHS 111 calls and GP contacts were lower in the molnupiravir arm than in the usual care arm during the first 28 days after randomisation. Those in the usual care arm were more likely to use NHS 111 (−0.024 contacts, 95% CI = −0.032 to −0.016, *P*<0.001) and contact their GPs for their conditions (−0.092 contacts, 95% CI = −0.12 to −0.067, *P*<0.001) than those in the molnupiravir arm. Those in the molnupiravir arm also reported fewer contacts with other types of services reported in the free-text entries than those in the usual care arm (−0.027 contacts, 95% CI = −0.043 to −0.011, *P* = 0.001).

Between 28 days and 3 months post-randomisation, participants in the molnupiravir arm reported fewer GP video consultations (−0.0043 contacts, 95% CI = −0.0084 to −0.0007, *P* = 0.026), practice nurse consultations (−0.012, 95% CI = −0.021 to −0.0031, *P* = 0.009), and less time off work (−0.30 days, 95% CI = −0.54 to −0.070, *P* = 0.010) than participants in the usual care arm.

Participants in the molnupiravir arm reported more respiratory outpatient visits (0.0076 contacts, 95% CI = 0.0021 to 0.013, *P* = 0.007) and more social worker visits (0.0007 contacts, 95% CI = 0.0002 to 0.0015, *P* = 0.033) than participants in the usual care arm, but fewer community nurse consultations (−0.0057 contacts, 95% CI = −0.0102 to −0.0020, *P* = 0.007), between 3 and 6 months post-randomisation (Supplementary Table S10).

The mean length of hospital stay was not statistically significantly different between the molnupiravir arm and usual care arm at the different time points (Supplementary Table S11).

### Costs

The mean cost of admission to hospital, which consisted of the cost of admitted patient care and critical care, was the main cost driver among the resource items across the time points in the available analysis. Overall, there were no statistically significant differences in mean NHS and PSS costs, or economic values associated with time off work between the treatment arms during each period of follow-up (see Supplementary Table S12). The exceptions were the mean cost of NHS 111 calls (−£2.1, 95% CI = −2.9 to −1.4, *P*<0.001), GP contacts (−£3.6, 95% CI = −4.6 to −2.6, *P*<0.001), and other types of services reported in the free-text entries (−£6.0, 95% CI = −9.8 to −2.5, *P* = 0.001), which were lower in the molnupiravir arm than in the usual care arm during the first 28 days after randomisation.

Between 28 days and 3 months post-randomisation, participants in the molnupiravir arm had a lower mean cost of GP video consultations (−£0.16, 95% CI = −0.32 to −0.027, *P* = 0.026), practice nurse consultations (−£0.08, 95% CI = −0.14 to −0.021, *P* = 0.009), and valuation of time off work (−£37, 95% CI = −66 to −9, *P* = 0.009) than participants in the usual care arm (Supplementary Table S12).

Between 3 and 6 months post-randomisation, participants in the molnupiravir arm incurred higher mean respiratory outpatient costs (£1.1, 95% CI = 0.24 to 2.0, *P* = 0.014) and social worker costs (£0.006, 95% CI = 0.0015 to 0.012, *P* = 0.033), but had lower community nurse consultation costs (−£0.039, 95% CI = −0.069 to −0.013, *P* = 0.007), compared with participants in the usual care arm (Supplementary Table S12).

### Health utilities

In the available case analysis (Supplementary Table S13) of EQ-5D-5L utility scores, participants in the molnupiravir arm had a higher mean EQ-5D-5L utility score than those in the usual care arm at 14 days (0.0087, 95% CI = 0.0038 to 0.013, *P* = 0.001) and 3 months (0.0066, 95% CI = 0.0014 to 0.012, *P* = 0.012) post-randomisation. There was no statistically significant difference in mean EQ-5D-5L utility scores between the treatment arms at 6 months post-randomisation (0.0033, 95% CI = −0.002 to 0.009, *P* = 0.24). The EQ-5D-5L VAS score was statistically significantly higher in the molnupiravir arm than the usual care arm (*P*≤0.0001) at all follow-up time points.

### Cost-effectiveness results

The incremental cost-effectiveness analysis results for molnupiravir versus usual care are presented in Table 1 for the base-case analysis using the imputed dataset and for each of the sensitivity, selected subgroup, and post hoc subgroup analyses. Table 1 also presents the probability that molnupiravir is cost-effective relative to usual care at different recommended cost-effectiveness thresholds. The remaining subgroup analyses are presented in Supplementary Table S14.

**Table 1.** Incremental cost-effectiveness of molnupiravir with usual care versus usual care over 6 months of base-case, sensitivity, selected subgroup, and post hoc subgroup analyses, in 2020/2021 £ prices

**Analysis category**	**Molnupiravir: usual care, *n***	**Mean cost (SE)**		**Incremental cost (bootstrap 95% CI)**	**Mean QALYs (SE)**		**Incremental QALYs (bootstrap 95% CI)**	**ICER, £/QALY**	**Probability of molnupiravir being cost-effective at specified cost-effectiveness threshold**		
		
		**Molnupiravir**	**Usual care**		**Molnupiravir**	**Usual care**			**£15 000**	**£20 000**	**£30 000**
**Base-case analysis^a^**	12 821:12 962	1808 (1.5)	1359 (1.5)	449 (445 to 453)	0.4136 (0.0004)	0.4080 (0.0004)	0.0055 (0.0044 to 0.0067)	81 190 (NE quad)	<0.001	<0.001	<0.001

**Sensitivity analyses^a^**											
Societal perspective	12 821:12 962	2202 (2.6)	1861 (2.6)	341 (334 to 348)	0.4136 (0.0004)	0.4080 (0.0004)	0.0055 (0.0044 to 0.0067)	61 714 (NE quad)	<0.001	<0.001	<0.001
Complete cases	7822:6984	1385 (2.8)	800 (3.0)	586 (578 to 594)	0.4197 (0.0006)	0.4180 (0.0006)	0.0017 (0.0001 to 0.0034)	340 986 (NE quad)	<0.001	<0.001	<0.001

**Subgroup analyses^a^**											
*Age, years, with* *80 years as cut-off*											
<80	12 562:12 690	1793 (1.3)	1340 (1.3)	454 (450 to 457)	0.4142 (0.0004)	0.4088 (0.0004)	0.0054 (0.0043 to 0.0065)	83 838 (NE quad)	<0.001	<0.001	<0.001
≥80	259:272	2524 (26.2)	2276 (27.3)	248 (176 to 322)	0.3812 (0.0038)	0.3715 (0.0036)	0.0097 (−0.0006 to 0.020)	25 521 (NE quad)	0.39	0.44	0.54

*Immune disorders*											
No	11 696:11 892	1730 (0.8)	1308 (0.8)	422 (419 to 424)	0.4168 (0.0004)	0.4106 (0.0004)	0.0063 (0.0052 to 0.0074)	67 052 (NE quad)	<0.001	<0.001	<0.001
Yes	1125:1070	2621 (2.2)	1927 (2.8)	694 (686 to 701)	0.3793 (0.0017)	0.3799 (0.0017)	−0.0006 (−0.0052 to 0.0042)	Dominated (NW quad)	<0.001	<0.001	<0.001

*Doses of vaccination*											
0	143:132	2197 (27.0)	1569 (29.4)	628 (550 to 704)	0.3760 (0.0037)	0.3750 (0.0042)	0.001 (−0.0099 to 0.012)	610 155 (NE quad)	0.012	0.022	0.052
1	87:88	2548 (46.7)	1576 (45.4)	972 (846 to 1097)	0.3760 (0.0059)	0.3734 (0.0049)	0.0026 (−0.013 to 0.018)	378 798 (NE quad)	0.020	0.028	0.053
2	519:458	2206 (14.6)	1759 (16.5)	448 (405 to 492)	0.3793 (0.0023)	0.3760 (0.0025)	0.0033 (−0.0033 to 0.0099)	134 388 (NE quad)	0.015	0.025	0.059
3	11 836:12 044	1772 (1.3)	1330 (1.3)	443 (439 to 446)	0.4163 (0.0004)	0.4101 (0.0004)	0.0062 (0.0051 to 0.0074)	70 839 (NE quad)	<0.001	<0.001	<0.001
≥4	236:240	2196 (26.4)	1871 (28.5)	325 (251 to 401)	0.3855 (0.0037)	0.3962 (0.0035)	−0.011 (−0.021 to −0.0007)	Dominated (NW quad)	0.091	0.077	0.059

*NHS priority category*											
Category 2: Aged ≥80 years	259:272	2524 (16.4)	2276 (16.6)	248 (201 to 294)	0.3812 (0.0039)	0.3715 (0.0037)	0.0097 (−0.0009 to 0.020)	25 521 (NE quad)	0.39	0.44	0.54
Category 3: Aged ≥75 and <80 years	540:577	1651 (4.9)	1432 (4.8)	219 (206 to 233)	0.4165 (0.0015)	0.4083 (0.0015)	0.0082 (0.0039 to 0.013)	26 787 (NE quad)	0.28	0.37	0.55

**Post hoc subgroup analysis^a^**	799:849	1934 (12.2)	1702 (12.1)	232 (199 to 265)	0.4050 (0.0017)	0.3965 (0.0016)	0.0085 (0.0039 to 0.013)	27 129 (NE quad)	0.26	0.36	0.55

a *All base-case, sensitivity, and subgroup analyses were adjusted using age, vaccination status, and comorbidity unless stated otherwise. Post hoc subgroup analysis and age groups were adjusted by vaccination status and comorbidity. NHS priority category was adjusted by age and vaccination status. Number of doses of vaccination was adjusted by age and comorbidity. ICER = incremental cost-effectiveness ratio. NE = North-East. NW = North-West. QALY = quality-adjusted life year. Quad = quadrant of the cost-effectiveness plane. SE = standard error.*

**Figure 1.**
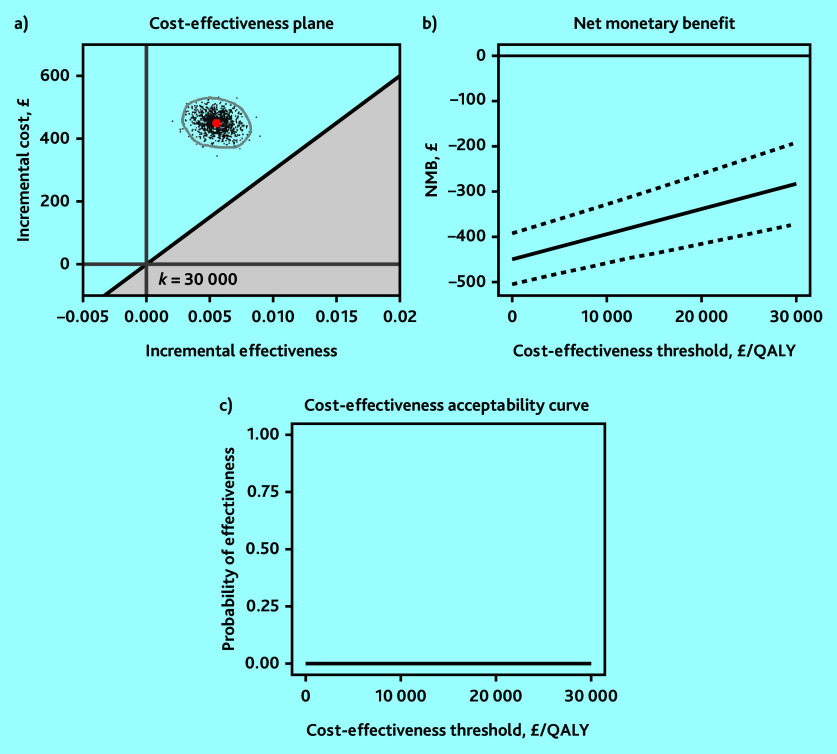
Base-case analysis of molnupiravir with usual care versus usual care using a) 95% confidence ellipse on the cost-effectiveness plane; b) net monetary benefit (NMB) with 95% confidence interval; and c) cost-effectiveness acceptability curve. QALY = quality-adjusted life year.

The base-case analysis showed that molnupiravir was not cost-effective relative to usual care at the recommended cost-effectiveness thresholds from an NHS and PSS perspective; participants in the molnupiravir arm had £449 (95% CI = 445 to 453) higher mean costs and generated 0.0055 (95% CI = 0.0044 to 0.0067) higher mean QALYs than usual care, resulting in a mean ICER of £81 190 per QALY gained (Table 1). The 95% confidence ellipse for the simulated ICER values fell above the upper range of the recommended cost-effectiveness threshold of £30 000 per QALY (Figure 1a) and its mean NMB was negative (Figure 1b). The probability of molnupiravir being cost-effective compared with usual care was zero at cost-effectiveness thresholds of £15 000, £20 000, and £30 000 per QALY (Figure 1c). Therefore, the base-case analysis indicated that molnupiravir was unlikely to be cost-effective relative to usual care.

Overall, this finding was robust to all sensitivity and subgroup analyses, which showed a similar finding that molnupiravir was not cost-effective relative to usual care over 6 months of follow-up (Table 1). In particular, those in the molnupiravir arm with immune disorders had higher mean costs (£694, 95% CI = 686 to 701) and similar mean QALYs (−0.0006, 95% CI = −0.0052 to 0.0042) than those in the usual care arm, hence ‘dominated’ in health economics terms. This finding was also observed among people that had ≥4 doses of vaccination as this group of people were likely to have immune disorders.

However, there was a 54% probability of molnupiravir being cost-effective relative to usual care for people aged ≥80 years at a cost-effectiveness threshold of £30 000 per QALY, as seen in the subgroup analysis by age group (NHS priority category 2, that is, aged ≥80 years) (Table 1).

Among those aged between 75 and 80 years (that is, NHS priority category 3), molnupiravir had a 55% probability of cost-effectiveness relative to usual care, assuming a £30 000 per QALY cost-effectiveness threshold. The post hoc subgroup analysis of participants aged ≥75 years showed that molnupiravir had a probability of 55% of being cost-effective relative to usual care at the £30 000 per QALY cost-effectiveness threshold (ICER £27 129 per QALY gained) (Table 1).

The post hoc subgroup analysis of participants aged ≥75 years showed that the main cost driver was admitted patient care during the first 28 days post-randomisation. Participants in the molnupiravir arm aged ≥75 years reported a statistically significant lower mean number of admitted patient care contacts (−0.032 contacts, 95% CI = −0.057 to −0.0087, *P* = 0.009) during the first 28 days post-randomisation and a statistically significant shorter mean length of hospital stay (−0.13 days, 95% CI = −0.25 to −0.029, *P* = 0.020) as shown in Supplementary Tables S4 and S5, respectively. This translated to a mean cost difference of −£131 (95% CI = −217 to −54, *P* = 0.002) for admitted patient care between those in the molnupiravir and usual care arms during the first 28 days (Supplementary Table S6). This finding represents an additional 26 admitted patients in the usual care arm during the first 28 days, so it is likely to be reasonably robust. Participants in the molnupiravir arm aged ≥75 years also had higher mean EQ-5D-5L utility and VAS scores at each stage of follow-up, although these differences were not statistically significant (Supplementary Table S7).

**Figure 2.**
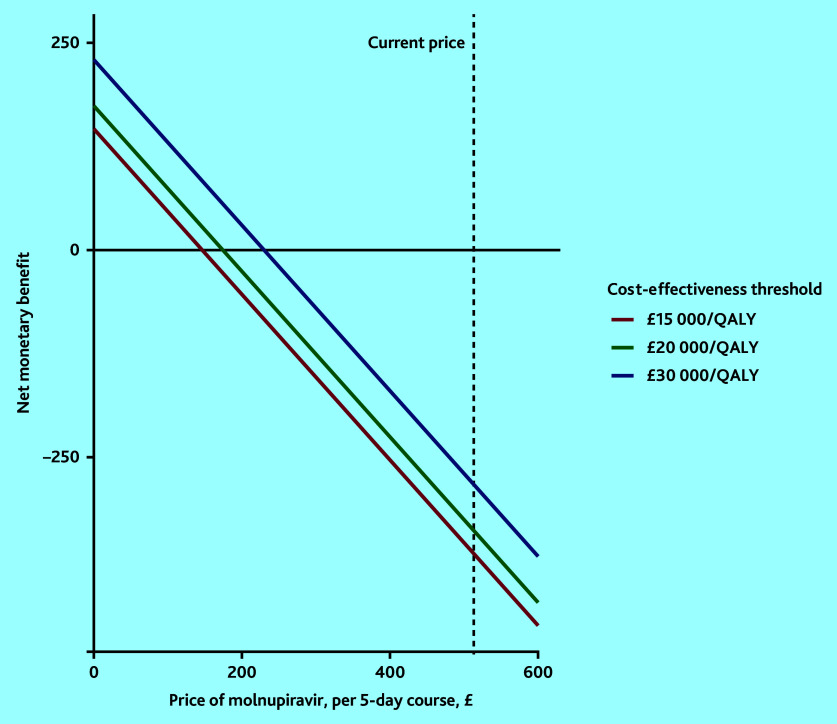
Threshold analysis of the price of a course of molnupiravir for it to be cost-effective at the range of cost-effectiveness thresholds. QALY = quality-adjusted life year.

The threshold analysis that investigated the acquisition price for molnupiravir at which it would be cost-effective is depicted in Figure 2. It showed that the price of molnupiravir would have to be set around £147, £174, or £230 per 5-day course to be cost-effective at cost-effectiveness thresholds of £15 000, £20 000, and £30 000 per QALY, respectively.

## Discussion

### Summary

The current analysis was based, to the authors’ knowledge, on the largest randomised trial yet, involving community-based people vaccinated against SARS-CoV-2 infection who are at increased risk of adverse COVID-19 outcomes and who were unwell with COVID-19. It showed that molnupiravir is unlikely to be cost-effective relative to usual care from either a UK NHS and PSS perspective or a UK societal perspective over the first 6 months after randomisation at an acquisition price of £513 per course. This finding was consistent in the sensitivity and subgroup analyses conducted. However, the analyses also showed that molnupiravir might be cost-effective relative to usual care among people aged ≥75 years if a cost-effectiveness threshold of £30 000 per QALY is adopted. The post hoc subgroup analysis showed that molnupiravir had a 55% probability of being cost-effective relative to usual care, likely supporting the treatment recommendation of the Australian Government Department of Health and Aged Care for people residing in residential aged care facilities.^33^

### Strengths and limitations

Although the economic evaluation was based on a large prospective, platform adaptive trial, which avoided many of the selection biases that characterised comparative studies,^34^^,^^35^ and included a ‘usual care’ comparator that restricted the potential for protocol-driven resource use, it is not without its limitations. First, and notably, the short time horizon of the trial that extended to 6 months’ post-randomisation. There is a possibility that the analyses failed to capture the economic consequences of long-term symptoms of COVID-19 and that longer-term follow-up of trial participants will rebalance the cost-effectiveness calculus. This may be the subject of future PANORAMIC analyses.

Second, it was assumed that the unit costs of resource inputs were applicable to all the nations of the UK because of limited nation-specific unit cost compendia available in the devolved nations. Third, resource-use and admission to hospital rates may be underestimated as patients with the highest risk of severe outcomes were excluded from this study for receiving treatment outside of this trial. Furthermore, existing economic tools did not allow the authors to value lost time among people who were not active in the labour market (for example, retired or unemployed). Given that molnupiravir was associated with less time off work, it is plausible that it also had positive effects on the use of leisure time and other time uses in those that were not in active employment. If this were the case, the cost-effectiveness estimates that are presented in this article should be viewed as conservative.

Next, further studies that are specifically targeted at older adults and that are adequately sized may be required to generate more precise estimates of cost-effectiveness than those presented here. Finally, the trial was open-label so differences in self-reported health status could be because of placebo effects.

There are two areas in this report that differed from the pre-specified HEAP. First, cost-effectiveness was not expressed in terms of incremental cost per admission to hospital or death prevented as this outcome is restricted to the first 28 days post-randomisation and the 28-day time horizon may not be long enough to capture the full benefits of molnupiravir, especially that of persisting symptoms. Second, as noted, a post hoc subgroup analysis of those aged ≥75 years was included following results from the pre-specified subgroup analyses indicating a likelihood that molnupiravir is cost-effective in specific older age groups identified as NHS priority categories.

### Comparison with existing literature

This is, to the authors’ knowledge, the first within-trial cost-utility analysis involving molnupiravir for the treatment of COVID-19. Previous studies examining the cost-effectiveness of molnupiravir versus standard care used decision-analytic modelling with contrasting results. Jo *et al*^35^ found that molnupiravir was unlikely to be cost-effective in terms of avoidance of hospital/intensive care unit admissions relative to standard care from the Korean health system perspective in a mainly unvaccinated population over 1 year. Wai *et al*^36^ found an incremental cost-effectiveness ratio of $493 345 (or £400 349 using an exchange rate of $1 = £0.8115 in 2022^37^) per death averted for molnupiravir versus standard care among patients with mild-to-moderate COVID-19 and unknown vaccination status in the outpatient setting over a 28-day time horizon. This would make molnupiravir unlikely to be cost-effective relative to standard care using the NICE recommended thresholds of £20 000 to £30 000 per QALY gained. In contrast, Goswami *et al*^34^ found that molnupiravir was likely to be cost-effective relative to standard care from the US payer perspective over a lifetime time horizon among an unvaccinated population. However, all studies included direct medical costs only whereas the current study encompassed direct medical costs, direct non-medical costs, and indirect costs incurred by patients with COVID-19. Furthermore, because of differences in vaccine coverage, and the organisation and delivery of health systems, the findings from the earlier studies are unlikely to be generalisable to the UK health system setting.

### Implications for research and practice

In conclusion, although the overall findings showed that molnupiravir is unlikely to be cost-effective in the studied population, there might be a subgroup of patients (that is, people aged ≥75 years) for whom molnupiravir is cost-effective. PANORAMIC is a platform trial that allows potentially competing treatments (for example, nirmatrelvir and ritonavir) to be added to the platform and their relative cost-effectiveness to be assessed. Findings from this study will help inform procurement strategies and influence policymaking around antiviral treatments for COVID-19. Incorporation of the economic consequences of longer-term persisting symptoms beyond the 6-month time horizon adopted by this study or a reduction in the market price of molnupiravir may widen the patient groups for whom molnupiravir is likely to be cost-effective.

## Data

Qualifying researchers who wish to access the data should submit a proposal with a valuable research question. Proposals will be assessed by a committee formed from the trial management group, including senior statistical and clinical representation. Data will be shared in accordance with the data sharing policy of Nuffield Department of Primary Care Health Sciences.
